# Supplementary material for: Very Low Alcohol Consumption Is Associated with Lower Prevalence of Cirrhosis and Hepatocellular Carcinoma in Patients with Non-Alcoholic Fatty Liver Disease
Source: Nutrients. 2022 Jun 16;14(12):2493. doi: 10.3390/nu14122493 (PMC9231078; doi:10.3390/nu14122493)
Supplement: Supplementary file 1 [file nutrients-14-02493-s001.zip › nutrients-1698245-supplementary.pdf]

Table S1: Binge drinkers characteristics according to current alcohol intake per week

| **Variable** | **Current abstainers n=3** | **Current drinkers n=32** | ***p*** | **C1 n=14** | **C2 n=18** | ***p*** |
|---|---|---|---|---|---|---|
| Age (years) | 75 (68-78) | 58 (18-88) | | 56 (32-71) | 68 (30-86) | |
| Male sex | 3 (100%) | 27 (84.3%) | | 13 (92.9%) | 13 (72.2%) | |
| Waist (cm) | 110 (100-119) | 101 (85-123) | | 101 (85-114) | 105 (87-123) | |
| BMI | 31.3 (28.4-37.2) | 28.1 (22.3-37.5) | | 27.2 (24.3-33.0) | 29.4 (22.3-37.5) | |
| Hypertension | 2 (66.7%) | 20 (62.5%) | | 9 (64.3%) | 11 (61.1%) | |
| DM type 2 | 0 (0%) | 7 (21.9%) | | 2 (14.3%) | 5 (27.8%) | |
| Dyslipidemia | 1 (33.3%) | 20 (62.5%) | | 7 (50.0%) | 13 (78.0%) | |
| Smoking habit, ever | 0 (0%) | 15 (46.9%) | | 6 (42.9%) | 9 (50.0%) | |
| Among smokers (p/y) | n.a. | 30 (3-90) | | 11.3 (3-20) | 30 (10-90) | |
| Current alcohol units/week | 0 | 7 (1-21) | *0.01* | 4 (2-6) | 16 (7-21) | *a,c* |
| LACU | 91.1 (30-137.1) | 30.7 (0.7-200.0) | | 17.1 (7.1-50.0) | 95 (10.0-200.0) | *a,c* |
| Drinking years | 30 (5-60) | 30 (1-60) | | 21 (15-55) | 35 (3-60) | |
| Exclusive wine drinkers | 0 (0%) | 10 (31.3%) | | 1 (7.1%) | 9 (50.0%) | *c* |
| Overall wine drinkers | 3 (100%) | 27 (84.4%) | | 10 (71.4%) | 17 (94.4%) | |
| Exclusive beer drinkers | 0 (0%) | 3 (9.4%) | | 2 (14.3%) | 1 (5.6%) | |
| Overall beer drinkers | 1 (33.3%) | 17 (53.1%) | | 8 (57.1%) | 9 (50.0%) | |
| Exclusive spirits drinkers | 0 | 2 (6.3%) | | 2 (14.3%) | 0 (0%) | |
| Overall spirits drinkers | 3 (100%) | 16 (50%) | | 7 (50.0%) | 9 (50.0%) | |
| Leisure physical activity | 1.0 ± 0.5 | 1.35 ± 0.68 | | 1.43 ± 0.69 | 1.25 ± 0.68 | |
| Coffee cups per day | 1 (0-2) | 2 (0-4) | | 2 (0-4) | 2 (0-3) | |
| Soft drinks per week | 1 (0-3) | 2 (0-5) | | 2 (0-5) | 2 (0-4) | |
| HbA1c (mmol/mol) | 40 (38-42) | 42 (33-60) | | 42 (35-45) | 45 (33-60) | |
| Total cholesterol (mg/dl) | 167 (134-184) | 195 (148-267) | | 201 (149-267) | 180 (148-234) | |
| HDL cholesterol (mg/dl) | 48 (31-58) | 45 (32-78) | | 43 (32-70) | 46 (37-78) | |
| LDL cholesterol (mg/dl) | 105 (101-109) | 119 (78-186) | | 133 (78-186) | 103 (82-140) | |
| Triglycerids (mg/dl) | 86 (49-123) | 104 (32-810) | | 117 (58-209) | 96 (32-287) | |
| Ferritin (ng/ml) | 147 (122-170) | 158 (23-358) | | 203 (23-358) | 158 (27-227) | |
| Urate (mg/dl) | 5.4 (5.2-5.6) | 5.7 (3.8-7.6) | | 6.4 (4.7-7.6) | 5.3 (3.8-6.9) | |
| AST (U/l) | 54 (36-68) | 31 (15-61) | *0.05* | 30 (15-61) | 32 (21-60) | |
| ALT (U/l) | 29 (22-43) | 37 (17-151) | | 40 (17-105) | 36 (19-151) | |
| Total bilirubin (mg/dl) | 0.9 (0.5-1.7) | 0.8 (0.3-2.0) | | 0.9 (0.6-1.7) | 0.8 (0.3-2.0) | |
| Platelet count (x$10^3$/μl) | 138 (94-186) | 196 (44-402) | *0.07* | 217 (144-391) | 183 (111-357) | |
| gammaGT (U/l) | 36 (18-141) | 53 (18-270) | | 54 (18-270) | 42 (18-179) | |
| Albumin (g/dl) | 3.5 (2.9-4.0) | 4.2 (2.6-4.9) | *0.06* | 4.2 (3.5-4.8) | 4.1 (3.0-4.5) | |
| HOMA (DM excluded) | 2.5 (1.2-3.7) | 2.8 (1.4-7.8) | | 2.5 (1.4-7.8) | 3.8 (2.1-6.2) | |
| NFS | 1.4 (+0.0/+2.8) | -1.9 (-4.4/+4.0) | *0.04* | -0.9(-4.4/+0.5) | -0.7(-4.1/+2.1) | |
| FIB-4 | 4.45 (2.67-6.22) | 1.40 (0.36-2.61) | *0.002* | 1.29 (0.36-2.61) | 1.38 (0.60-5.89) | *a* |
| HSI | 37.6 (33.3-40.6) | 40.3 (30.0-61.6) | | 40.2 (36.5-46.4) | 40.2 (32.5-48.4) | |
| Subcutaneous fat (mm) | 19 (15-22) | 17 (10-85) | | 17 (10-47) | 17 (10-85) | |
| Visceral fat (mm) | 73 (50-95) | 69 (25-116) | | 86 (54-120) | 67 (25-116) | |
| 2D-SWE (kPa) | 15.2 (10.2-20.4) | 6.7 (5.3-20.1) | | 6.0 (5.3-7.8) | 8.5 (5.6-20.1) | *a,c* |
| Cirrhosis | 2 (66.7%) | 5 (15.6%) | *0.03* | 0 (0%) | 5 (27.8%) | *a,c* |
| HCC | 1 (33.3%) | 5 (15.6%) | | 0 (0%) | 5 (27.8%) | *c* |

Active drinkers were divided in C1 (very low consumers, < 70 grams per week), C2 (moderate consumers, 70-210 grams per week for men and 70-140 grams per week for women).

Data are expressed as median (range) or absolute number (%) except for leisure physical activity which is expressed as mean ± standard deviation. Only statistically significant differences are reported in the p columns. In p column, *a* means p < 0.0167 between current abstainers and C1, *b* means p < 0.0167 between current abstainers and C2, *c* means p < 0.0167 between C1 and C2.

Table S2: Patient characteristics according to current alcohol intake per week (binge drinkers excluded)

| Variable | Current abstainers n=89 | Current drinkers n=152 | *p* | C1 n=103 | C2 n=49 | *p* |
|---|---|---|---|---|---|---|
| Age (years) | 61 (18-85) | 58 (18-88) | | 56 (18-78) | 68 (18-88) | *c* |
| Male sex | 32 (36.0%) | 101 (66.4%) | *<0.001* | 66 (64.1%) | 35 (71.4%) | *a, b* |
| Waist (cm) | 104 (76-160) | 104 (60-142) | | 103 (75-124) | 105 (60-142) | |
| BMI | 29.1 (20.1-44.0) | 29.0 (18.6-48.8) | | 29.4 (20.8-42.2) | 27.8 (18.6-48.8) | |
| Hypertension | 49 (55.1%) | 87 (57.2%) | | 54 (52.4%) | 33 (67.3%) | |
| DM type 2 | 30 (33.3%) | 37 (24.3%) | | 21 (20.4%) | 16 (32.7%) | |
| Dyslipidemia | 61 (68.9%) | 96 (63.2%) | | 64 (61.1%) | 32 (65.3%) | |
| Smoking habit, ever | 35 (38.9%) | 78 (51.3%) | | 48 (46.6%) | 30 (61.2%) | |
| Among smokers (p/y) | 15.6 (0.5-100) | 16.5 (0.5-100) | | 15 (1-90) | 22 (0.5-100) | |
| Current alcohol units/week | 0 | 3 (1-21) | *<0.001* | 2 (1-6) | 12 (7-21) | *c* |
| LACU | 0 (0-160.7) | 10.4 (0.7-200.0) | *<0.001* | 5.7 (0.7-122.9) | 40 (3.0-200.0) | *b, c* |
| Drinking years | 0 (0-60) | 20 (1-60) | *<0.001* | 20 (1-55) | 30 (3-60) | *a,b,c* |
| Exclusive wine drinkers | 5 (5.6%) | 61 (40.1%) | *<0.001* | 40 (38.8%) | 21 (42.9%) | *a,b* |
| Overall wine drinkers | 11 (12.3%) | 125 (82.2%) | *<0.001* | 77 (74.8%) | 48 (98.0%) | *a,b,c* |
| Exclusive beer drinkers | 3 (3.2%) | 21 (13.8%) | *0.01* | 21 (20.4%) | 0 (0.0%) | *a* |
| Overall beer drinkers | 8 (9.0%) | 66 (43.4%) | *<0.001* | 53 (51.5%) | 13 (26.5%) | *a,b,c* |
| Exclusive spirits drinkers | 0 | 5 (3.3%) | | 4 (5.1%) | 1 (2.0%) | |
| Overall spirits drinkers | 4 (4.5%) | 38 (25%) | *<0.001* | 15 (14.6%) | 23 (46.9%) | *b,c* |
| Leisure physical activity | 1.15 ± 0.76 | 1.29 ± 0.68 | | 1.33 ± 0.69 | 1.19 ± 0.68 | |
| Coffee cups per day | 2 (0-6) | 2 (0-9) | *0.04* | 2 (0-9) | 2 (0-7) | |
| Soft drinks per week | 2 (0-6) | 2 (0-7) | | 2 (0-6) | 2 (0-7) | |
| HbA1c (mmol/mol) | 48 (23-109) | 40 (25-94) | | 40 (34-94) | 45 (25-103) | |
| Total cholesterol (mg/dl) | 198 (69-353) | 194 (84-328) | | 188 (120-256) | 204 (84-309) | |
| HDL cholesterol (mg/dl) | 50 (29-119) | 47 (11-86) | *0.02* | 46 (29-86) | 48 (11-84) | |
| LDL cholesterol (mg/dl) | 129 (55-231) | 122 (50-208) | | 115 (55-184) | 138 (50-201) | |
| Triglycerids (mg/dl) | 114 (35-934) | 124 (32-810) | | 124 (44-810) | 141 (32-667) | |
| Ferritin (ng/ml) | 66 (7-876) | 115 (7-2098) | *0.04* | 87 (7-349) | 165 (7-2098) | |
| Urate (mg/dl) | 5.8 (2.5-7.7) | 6.0 (2.5-10.2) | | 6.0 (2.5-10.2) | 5.8 (3.8-6.6) | |
| AST (U/l) | 32 (13-200) | 29 (11-200) | | 30 (18-200) | 26 (11-80) | |
| ALT (U/l) | 37 (8-230) | 33 (11-275) | | 38 (15-275) | 28 (11-152) | |
| Total bilirubin (mg/dl) | 0.7 (0.3-2.8) | 0.7 (0.3-3.0) | | 0.7 (0.3-1.6) | 0.8 (0.3-3.0) | |
| Platelet count ($x10^3/\mu l$) | 224 (48-400) | 228 (44-402) | | 237 (44-402) | 218 (81-393) | |
| gammaGT (U/l) | 52 (8-905) | 50 (15-410) | | 52 (18-410) | 46 (15-406) | |
| Albumin (g/dl) | 4.1 (2.9-5.0) | 4.1 (2.6-4.9) | | 4.2 (3.2-4.7) | 4.1 (2.6-4.9) | |
| HOMA (DM excluded) | 2.5 (1.4-8.9) | 2.1 (0.5-11.6) | | 1.8 (1.4-5.2) | 3.8 (0.5-11.6) | *c* |
| NFS | -1.3 (-3.6/+2.8) | -1.2 (-4.6/+4.0) | | -1.9(-4.6/+4.0) | -0.7(-4.4/+2.6) | *c* |
| FIB-4 | 1.28 (0.31-12,74) | 1.19 (0.19-8.5) | | 1.00 (0.23-8.5) | 1.40 (0.19-6.3) | |
| HSI | 41.3 (30.1-57.8) | 40.5 (30.0-61.6) | | 40.9 (31.8-61.6) | 38.9 (30.0-56.6) | |
| Subcutaneous fat (mm) | 20 (10-60) | 20 (10-85) | | 20 (10-47) | 20 (10-85) | |
| Visceral fat (mm) | 68 (34-126) | 70 (15-179) | | 67 (15-120) | 73 (33-179) | |
| 2D-SWE (kPa) | 6.8 (3.7-27.2) | 6.2 (3.3-30.2) | | 6.0 (3.3-22.1) | 9.6 (3.5-50.1) | *a,b,c* |
| Cirrhosis | 24 (27.0%) | 25 (16.4%) | *0.05* | 12 (11.7%) | 13 (26.5%) | *a,c 0.02* |
| HCC | 11 (12.2%) | 15 (9.9%) | | 4 (3.9%) | 11 (22.4%) | *a 0.03,c* |

Current abstainers include 17 ex-drinkers. Active drinkers were divided in C1 (very low consumers, < 70 grams per week), C2 (moderate consumers, 70-210 grams per week for men and 70-140 grams per week for women).

Data are expressed as median (range) or absolute number (%) except for leisure physical activity which is expressed as mean ± standard deviation. Only statistically significant differences are reported in the p columns. In p column, *a* means p < 0.0167 between current abstainers and C1, *b* means p < 0.0167 between current abstainers and C2, *c* means p < 0.0167 between C1 and C2.

Table S3: Binge drinkers characteristics according to lifetime alcohol exposure (LACU). LACU aggregate.

| **Variable** | **Abstainers n=75** | **Consumers n=35** | ***p*** | **Q1-Q3 n=18** | **Q4 n=17** | ***p*** |
|---|---|---|---|---|---|---|
| Age (years) | 59 (18-80) | 62 (18-88) | | 49 (32-83) | 73 (49-86) | *b,c* |
| Male sex | 25 (33.3%) | 29 (82.9%) | *<0.001* | 16 (88.9%) | 13 (76.5%) | *a,b* |
| Waist (cm) | 103 (88-160) | 101 (81-123) | | 101 (81-123) | 100 (85-122) | |
| BMI | 28.8 (24.2-44.0) | 28.2 (18.6-48.8) | | 27.6 (18.6-48.8) | 28.9 (23.2-40.0) | |
| Hypertension | 43 (56.7%) | 22 (62.9%) | | 11 (61.1%) | 11 (64.7%) | |
| DM type 2 | 26 (35.7%) | 7 (20.0%) | | 2 (11.1%) | 5 (29.4%) | |
| Dyslipidemia | 52 (69,3%) | 20 (57.1%) | | 9 (50.0%) | 11 (64.7%) | |
| Smoking habit, ever | 28 (36.8%) | 17 (48.6%) | | 9 (50.0%) | 8 (47.1%) | |
| Among smokers p/y | 20.0 (0.5-90) | 20 (3-90) | | 11 (3-30) | 30 (10-90) | |
| Current alcohol units/week | 0 | 7 (0-21) | *<0.001* | 4 (0-21) | 15 (0-21) | *a,b,c* |
| LACU | 0 | 31.4 (6.0-200.0) | *<0.001* | 17.1 (6.0-31.4) | 110 (42.8-200.0) | *a,b,c* |
| Drinking years | 0 | 30 (3-60) | *<0.001* | 20 (3-55) | 45 (5-60) | *a,b,c* |
| Exclusive wine drinkers | 0 | 7 (20%) | | 1 (40.4%) | 6 (36.7%) | |
| Overall wine drinkers | 0 | 29 (82.9%) | | 13 (78.7%) | 16 (96.7%) | |
| Exclusive beer drinkers | 0 | 4 (11.4%) | | 4 (22.2%) | 0 (0%) | |
| Overall beer drinkers | 0 | 18 (51.4%) | | 12 (66.7%) | 6 (35.3%) | |
| Exclusive spirits drinkers | 0 | 2 (5.7%) | | 1 (5.6%) | 1 (5.9%) | |
| Overall spirits drinkers | 0 | 19 (54.3%) | | 9 (50.0%) | 10 (58.8%) | |
| Leisure physical activity | 1.20 ± 0.80 | 1.30 ± 0.68 | | 1.44 ± 0.72 | 1.09 ± 0.71 | |
| Coffee cups per day | 2 (0-6) | 2 (0-4) | | 2 (0-4) | 2 (1-3) | |
| Soft drinks per week | 2 (0-6) | 2 (0-5) | | 2 (0-5) | 2 (0-4) | |
| HbA1c (mmol/mol) | 49 (23-109) | 42 (33-60) | | 39 (33-45) | 42 (37-60) | |
| Total cholesterol (mg/dl) | 196 (69-353) | 193 (149-267) | | 197 (149-267) | 182 (149-208) | |
| HDL cholesterol (mg/dl) | 49 (29-76) | 46 (31-78) | | 44 (31-78) | 50 (37-70) | |
| LDL cholesterol (mg/dl) | 128 (55-231) | 114 (32-208) | | 119 (78-186) | 106 (82-140) | |
| Triglycerides (mg/dl) | 114 (42-934) | 104 (32-287) | | 116 (32-209) | 79 (49-287) | |
| Ferritin (ng/ml) | 65 (7-876) | 158 (23-761) | *0.05* | 131 (23-761) | 165 (150-227) | |
| Urate (mg/dl) | 5.7 (2.5-7.7) | 5.5 (2.5-10.2) | | 7.1 (6.1-7.6) | 5.2 (3.8-6.9) | *c* |
| AST (U/l) | 32 (13-200) | 34 (15-68) | | 34 (15-61) | 34 (19-68) | |
| ALT (U/l) | 37 (8-230) | 36 (13-275) | | 42 (17-105) | 33 (19-151) | |
| Total bilirubin (mg/dl) | 0.67 (0.3-2.8) | 0.86 (0.6-2.0) | | 0.96 (0.6-2.0) | 0.75 (0.6-1.7) | |
| Platelet count (x$10^3$/μl) | 224 (48-400) | 188 (44-358) | | 207 (94-358) | 111 (44-276) | *c* |
| gammaGT (U/l) | 52 (8-905) | 53 (18-270) | | 52 (18-270) | 55 (18-124) | |
| Albumin (g/dl) | 4.1 (2.9-4.8) | 4.1 (2.6-4.8) | | 4.2 (3.5-4.8) | 4.0 (2.9-4.7) | |
| HOMA (DM excluded) | 1.9 (1.4-8.9) | 2.8 (1.4-7.8) | | 2.5 (1.4-7.8) | 3.8 (2.1-6.2) | |
| NFS | -1.1 (-3.6/+1.1) | -0.8 (-4.1/+2.8) | | -2.4 (-4.1/+0.5) | 0.0 (-2.6/+2.8) | *b, c* |
| FIB-4 | 1.45 (0.3-12.7) | 1.52 (0.4-6.2) | | 1.1 (0.4-2.6) | 1.9 (0.9-6.2) | *b* |
| HSI | 40.4 (30.0-53.8) | 39.9 (30.1-61.6) | | 38.8 (30.1-61.6) | 40.6 (33.0-50.6) | |
| Subcutaneous fat (mm) | 21 (15-60) | 17 (10-85) | *0.002* | 16 (10-30) | 18 (12-22) | *a* |
| Visceral fat (mm) | 67 (35-110) | 69 (21-126) | | 86 (12-120) | 64 (36-116) | |
| 2D-SWE (kPa) | 6.8 (3.7-27.2) | 7.0 (3.3-50.5) | | 6.0 (5.0-15.2) | 9.6 (7.0-36.1) | *c* |
| Cirrhosis | 21 (27.6%) | 7 (20.0%) | | 1 (5.6%) | 6 (35.3%) | |
| HCC | 11 (14.5%) | 6 (17.1%) | | 0 (0%) | 6 (35.3%) | *c* |

Q1-3 (<4.29-40.00 LACU), Q4 (>40.01 LACU).

Data are expressed as median (range) or absolute number (%) except for leisure physical activity which is expressed as mean ± standard deviation. Only statistically significant differences are reported in the p columns. In p column, *a* means p < 0.0167 between abstainers and Q1-Q3, *b* means p < 0.0167 between abstainers and Q4, *c* means p < 0.0167 between Q1-3 and Q4.

Table S4: Patient characteristics according to lifetime alcohol exposure (LACU) (binge drinkers excluded). LACU aggregate.

| Variable | Abstainers n=75 | Consumers n=166 | *p* | Q1-Q3 n=136 | Q4 n=30 | *p* |
|---|---|---|---|---|---|---|
| Age (years) | 59 (18-80) | 58 (18-88) | | 56 (18-78) | 69 (45-86) | *b, c* |
| Male sex | 25 (33.3%) | 108 (65.1%) | *<0.001* | 85 (62.5%) | 23 (76.7%) | *a, b* |
| Waist (cm) | 103 (88-160) | 105 (60-142) | | 104 (60-142) | 112 (85-142) | |
| BMI | 28.8 (24.2-44.0) | 29.1 (18.6-48.8) | | 29.4 (18.6-48.8) | 28.0 (23.2-40.0) | |
| Hypertension | 43 (56.7%) | 93 (57.2%) | | 70 (51.5%) | 23 (76.7%) | |
| DM type 2 | 26 (35.7%) | 41 (24.7%) | | 31 (22.8%) | 10 (33.3%) | |
| Dyslipidemia | 52 (69,3%) | 106 (63.8%) | | 86 (63.2%) | 20 (66.7%) | |
| Smoking habit, ever | 28 (36.8%) | 83 (50.0%) | *0.05* | 66 (48.5%) | 17 (56.7%) | |
| Among smokers p/y | 20.0 (0.5-90) | 16.0 (0.5-100) | | 15.0 (0.5-100) | 30.0 (3-100) | |
| Current alcohol units/week | 0 | 3 (0.5-21.0) | *<0.001* | 2 (0-21) | 14 (0-21) | *a,b,c* |
| LACU | 0 | 10.0 (0.7-200.0) | *<0.001* | 7.1 (0.7-40.0) | 77.0 (42.8-200.0) | *a,b,c* |
| Drinking years | 0 | 20 (1-60) | *<0.001* | 20 (1-60) | 40 (20-60) | *a,b,c* |
| Exclusive wine drinkers | 0 | 66 (39.8%) | | 55 (40.4%) | 11 (36.7%) | |
| Overall wine drinkers | 0 | 136 (81.9%) | | 107 (78.7%) | 29 (96.7%) | |
| Exclusive beer drinkers | 0 | 24 (14.4%) | | 24 (17.6%) | 0 | |
| Overall beer drinkers | 0 | 74 (44.6%) | | 68 (50.0%) | 6 (20%) | *c* |
| Exclusive spirits drinkers | 0 | 5 (3.0%) | | 4 (2.9%) | 1 (3.3%) | |
| Overall spirits drinkers | 0 | 42 (25.3%) | | 22 (16.2%) | 20 (66.7%) | *c* |
| Leisure physical activity | 1.20 ± 0.80 | 1.23 ± 0.68 | | 1.25 ± 0.72 | 1.20 ± 0.71 | |
| Coffee cups per day | 2 (0-6) | 2 (0-9) | | 2 (0-9) | 2 (0-4) | |
| Soft drinks per week | 2 (0-6) | 2 (0-7) | | 2 (0-7) | 2 (0-6) | |
| HbA1c (mmol/mol) | 49 (23-109) | 41 (27-103) | *0.04* | 41 (27-103) | 37 (28-85) | |
| Total cholesterol (mg/dl) | 196 (69-353) | 195 (84-328) | | 194 (84-309) | 195 (99-328) | |
| HDL cholesterol (mg/dl) | 49 (29-76) | 48 (11-119) | | 47 (12-86) | 53 (11-119) | |
| LDL cholesterol (mg/dl) | 128 (55-231) | 122 (32-208) | | 119 (32-201) | 131 (51-208) | |
| Triglycerides (mg/dl) | 114 (42-934) | 124 (32-810) | | 124 (32-810) | 128 (49-287) | |
| Ferritin (ng/ml) | 65 (7-876) | 99 (7-2098) | | 89 (7-761) | 266 (20-2098) | *b, c* |
| Urate (mg/dl) | 5.7 (2.5-7.7) | 6.1 (2.5-10.2) | | 6.3 (2.5-10.2) | 5.2 (3.8-7.4) | *c* |
| AST (U/l) | 32 (13-200) | 29 (11-200) | | 30 (14-200) | 25 (11-92) | |
| ALT (U/l) | 37 (8-230) | 33 (13-275) | | 37 (13-275) | 27 (15-151) | |
| Total bilirubin (mg/dl) | 0.67 (0.3-2.8) | 0.71 (0.4-3.0) | | 0.70 (0.3-3.0) | 0.76 (0.4-2.3) | |
| Platelet count ($x10^3/\mu l$) | 224 (48-400) | 228 (44-402) | | 241 (44-402) | 205 (44-298) | *c* |
| gammaGT (U/l) | 52 (8-905) | 50 (16-410) | | 47 (12-410) | 63 (18-301) | |
| Albumin (g/dl) | 4.1 (2.9-4.8) | 4.1 (2.6-4.9) | | 4.1 (2.6-4.9) | 4.0 (2.9-4.7) | *c* |
| HOMA (DM excluded) | 1.9 (1.4-8.9) | 2.3 (0.5-11.6) | | 2.3 (0.5-11.6) | 2.8 (1.8-3.8) | |
| NFS | -1.1 (-3.6/+1.1) | -1.2 (-4.6/+4.0) | | -1.8 (-4.6/+4.0) | 0.3 (-2.7/+2.8) | *b, c* |
| FIB-4 | 1.45 (0.3-12.7) | 1.19 (0.2-8.5) | | 0.99 (0.2-8.5) | 1.8 (0.7-6.3) | *c* |
| HSI | 40.4 (30.0-53.8) | 40.8 (30.1-61.6) | | 40.8 (30.1-61.6) | 40.8 (33.0-50.6) | |
| Subcutaneous fat (mm) | 21 (15-60) | 20 (10-85) | | 20 (10-85) | 20 (10-27) | |
| Visceral fat (mm) | 67 (35-110) | 72 (21-126) | | 71 (21-126) | 74 (36-116) | |
| 2D-SWE (kPa) | 6.8 (3.7-27.2) | 6.3 (3.3-50.5) | | 6.0 (3.3-50.5) | 16.0 (5.5-40.8) | *b, c* |
| Cirrhosis | 21 (27.6%) | 28 (16.9%) | *0.05* | 18 (13.2%) | 10 (33.3%) | *a, c* |
| HCC | 11 (14.5%) | 15 (9.0%) | | 4 (2.9%) | 11 (36.7%) | *a,b, c* |

Q1-3 (<4.29-40.00 LACU), Q4 (>40.01 LACU).
Data are expressed as median (range) or absolute number (%) except for leisure physical activity which is expressed as mean ± standard deviation. Only statistically significant differences are reported in the p columns. In p column, *a* means p < 0.0167 between abstainers and Q1-Q3, *b* means p < 0.0167 between abstainers and Q4, *c* means p < 0.0167 between Q1-3 and Q4.

**Table S5**: Patient characteristics according to lifetime alcohol exposure (LACU). All LACU subgroups.

| **Variable** | **Abstainers n=75** | **Consumers 201** | ***p*** | **Q1 n=56** | **Q2 n=48** | **Q3 n=50** | **Q4 n=47** | ***p*** |
|---|---|---|---|---|---|---|---|---|
| Age at first visit (years) | 59 (18-80) | 58 (18-88) | | 48 (18-78) | 54 (30-82) | 59 (18-88) | 71 (45-86) | *a,b,d,f,g* |
| Male sex | 23 (30.2%) | 137 (68.2%) | *<0.001* | 32 (57.1%) | 31 (64.6%) | 38 (76.0%) | 36 (76.6%) | *c,e,g* |
| Waist (cm) | 103 (88-160) | 104 (60-142) | | 103 (75-124) | 103 (60-142) | 105 (81-139) | 109 (85-142) | |
| BMI | 28.8 (24.2-44.0) | 29.1 (18.6-48.8) | | 29.1 (20.1-42.2) | 29.3 (18.6-48.8) | 29.1 (20.8-39.7) | 28.4 (23.2-40.0) | |
| Hypertension | 43 (56.7%) | 115 (57.2%) | | 28 (50.0%) | 22 (39.3%) | 31 (60.0%) | 34 (72.3%) | |
| DM type 2 | 26 (35.7%) | 48 (23.9%) | *0.03* | 13 (23.2%) | 9 (16.1%) | 11 (22.0%) | 15 (31.9%) | |
| Dyslipidemia | 52 (69,3%) | 126 (62.7%) | | 34 (60.7%) | 29 (60.4%) | 32 (64%) | 31 (66.0%) | |
| Smoking habit, ever | 28 (36.8%) | 100 (49,8%) | *0.04* | 25 (44.6%) | 27 (56.3%) | 24 (50.0%) | 24 (51,1%) | |
| Among smokers p/y | 20.0 (0.5-90) | 17.9 (0.5-100) | | 15.6 (0.5-90) | 10.6 (1-40) | 22.5 (5-100) | 30.0 (3-100) | *d* |
| Current alcohol units/week | 0 | 3.0 (0.5-21.0) | *<0.001* | 1 (0-14) | 3 (0-14) | 6 (0-21) | 14 (0-21) | * |
| LACU | 0 | 12.9 (0.7-200.0) | *<0.001* | 2.9 (0.7-4.3) | 8.6 (5.6-12.9) | 27.1 (14.3-40.0) | 80.0 (42.8-200) | * |
| Drinking years | 0 | 24 (1-60) | | | | | | |
| Binge-drinkers | 0 | 35 (17.4%) | | 2 (3.6%) | 7 (14.6%) | 9 (18.0%) | 17 (36.2%) | *b* |
| Excusive. wine drinkers | 0 | 73 (36.3%) | | 14 (25.0%) | 16 (33.3%) | 25 (50.0%) | 18 (38.3%) | |
| Overall wine drinkers | 0 | 165 (82.1%) | | 34 (60.7%) | 43 (89.6%) | 43 (86.0%) | 45 (95.7%) | |
| Excusive beer drinkers | 0 | 28 (13.9%) | | 19 (16.1%) | 3 (6.3%) | 6 (12%) | 0 | |
| Overall beer drinkers | 0 | 93 (46.3%) | | 32 (57.1%) | 28 (58.3%) | 19 (38%) | 14 (29.8%) | |
| Exclusive spirits drinkers | 0 | 7 (3.5%) | | 2 (3.6%) | 2 (4.2%) | 1 (2.0%) | 2 (4.1%) | |
| Overall spirits drinkers | 0 | 59 (29.4%) | | 6 (10.7%) | 11 (22.9%) | 10 (20.0%) | 32 (68%) | |
| Leisure physical activity | 1.20 ± 0.80 | 1.27 ± 0.68 | | 1.30 ± 0.72 | 1.34 ± 0.61 | 1.23 ± 0.69 | 1.17 ± 0.71 | |
| Coffee cups per day | 2 (0-6) | 2 (0-9) | | 2 (0-9) | 2 (0-7) | 2 (0-9) | 2 (0-4) | |
| Soft drinks servings/week | 2 (0-6) | 2 (0-7) | | 2 (0-7) | 2 (0-5) | 2 (0-7) | 2 (0-6) | |
| HbA1c (mmol/mol) | 49 (23-109) | 41 (27-103) | *0.02* | 39 (27-94) | 41 (29-103) | 45 (25-60) | 38 (28-85) | *a* |
| Total cholesterol (mg/dl) | 196 (69-353) | 195 (84-328) | | 197 (84-309) | 199 (98-267) | 194 (87-252) | 195 (99-328) | |
| HDL cholesterol (mg/dl) | 49 (29-76) | 48 (11-119) | | 47 (12-86) | 45 (30-84) | 48 (31-78) | 51 (11-119) | |
| LDL cholesterol (mg/dl) | 128 (55-231) | 119 (32-208) | | 121 (36-196) | 122 (50-186) | 116 (32-201) | 126 (51-208) | |
| Triglycerids (mg/dl) | 114 (42-934) | 123 (32-810) | | 124 (44-810) | 123 (55-408) | 119 (35-303) | 124 (49-287) | |
| Ferritin (ng/ml) | 65 (7-876) | 115 (7-2098) | *0.02* | 84 (7-349) | 131 (20-587) | 75 (7-761) | 244 (20-2098) | *g* |
| Urate (mg/dl) | 5.7 (2.5-7.7) | 5.9 (2.5-10.2) | *0.02* | 5.9 (2.5-10.2) | 6.5 (4.5-8.5) | 6.8 (4.7-9.5) | 5.2 (3.8-7.4) | *d,f* |
| urea (mg/dl) | 35 (11-52) | 36 (21-101) | | 32 (21-101) | 33 (21-59) | 32 (23-42) | 38 (21-49) | |
| AST (U/l) | 32 (13-200) | 30 (11-200) | | 31 (16-200) | 29 (14-83) | 30 (15-66) | 28 (11-92) | |
| ALT (U/l) | 37 (8-230) | 34 (13-275) | | 45 (15-275) | 34 (13-98) | 36 (13-134) | 29 (15-151) | |
| Total bilirubin (mg/dl) | 0.67 (0.3-2.8) | 0.71 (0.4-3.0) | | 0.75 (0.3-3.0) | 0.88 (0.4-2.0) | 0.66 (0.3-1.7) | 0.76 (0.4-2.3) | |
| Platelet count | 224 (48-400) | 223 (44-402) | | 242 (44-352) | 233 (106-402) | 251 (94-391) | 202 (44-298) | *f* |
| gammaGT (U/l) | 52 (8-905) | 51 (16-410) | | 53 (12-410) | 40 (15-406) | 50 (17-294) | 57 (18-301) | |
| Albumin (g/dl) | 4.1 (2.9-4.8) | 4.1 (2.9-4.9) | | 4.1 (3.2-4.7) | 4.3 (3.5-4.9) | 4.1 (2.6-4.9) | 4.0 (2.9-4.7) | *d* |
| HOMA (DM excluded) | 1.9 (1.4-8.9) | 2.4 (0.8-11.6) | | 2.9 (1.4-11.6) | 1.7 (0.5-6.0) | 2.7 (2.1-4.8) | 3.8 (1.8-6.2) | |
| NFS | -1.1 (-3.6/+1.1) | -1.1 (-4.6/+4.0) | | -2.0 (-3.7/+4.0) | -1.9 (-4.4/+2.6) | -1.2 (-4.6/+1.9) | 0.2 (-2.7/+2.8) | *b,d,f* |
| FIB-4 | 1.45 (0.31-12.74) | 1.21 (0.19-8.50) | | 0.97 (0.19-8.50) | 0.96 (0.23-4.46) | 1.06 (0.29-3.47) | 1.79 (0.74-6.30) | *d,f* |
| HSI | 40.4 (30.0-53.8) | 40.6 (30.1-61.6) | | 40.6 (30.1-61.6) | 40.3 (30.0-53.4) | 41.1 (32.5-56.6) | 40.6 (33.0-50.6) | |
| Subcutaneous fat (mm) | 21 (15-60) | 19 (10-85) | *0.02* | 20 (10-47) | 21 (10-45) | 19 (10-85) | 20 (10-27) | |
| Visceral fat (mm) | 67 (35-110) | 71 (21-126) | | 70 (21-120) | 65 (22-126) | 79 (21-123) | 72 (36-116) | |
| 2D-SWE (kPa) | 6.8 (3.7-27.2) | 6.5 (3.3-50.5) | | 6.5 (3.3-22.0) | 6.0 (3.5-34.0) | 6.2 (4.1-50.5) | 12.0 (5.5-40.8) | *b,d,g* |
| Cirrhosis | 21 (27.6%) | 35 (17.4%) | *0.05* | 7 (12.5%) | 6 (12.5%) | 6 (12.0%) | 16 (34.0%) | *b,f* |
| HCC | 11 (14.5%) | 21 (10.4%) | | 1 (1.8%) | 1 (2.1%) | 2 (4.0%) | 17 (36.2%) | *b,d,f,g* |

In p column, *a* means p < 0.005 between Q1 and abstainers, *b* means p < 0.005 between Q1 and Q4, *c* means p < 0.005 between Q2 and abstainers, *d* means p < 0.005 between Q2 and Q4, *e* means p < 0.005 between Q3 and abstainers, *f* means p < 0.005 between Q3 and Q4, *g* means p < 0.005 between Q4 and abstainers, * means p < 0.005 between all LACU categories.
